# An investigation into the avoidability of drug reaction with eosinophilia and systemic symptoms (DRESS) syndrome

**DOI:** 10.1038/s41598-021-97381-6

**Published:** 2021-09-09

**Authors:** Mohammed Ibn-Mas’ud Danjuma, Lina Mohammad Ahmad Naseralallah, Bodoor AbouJabal, Mouhand Faisal Mohamed, Ibrahim Y. Abubeker, Layla Abdul Jabbar, Abdelnaser Elzouki

**Affiliations:** 1 Department of Internal Medicine, Weill Cornell College of Medicine, New York, USA; 2 Department of Internal Medicine, Weill Cornell College of Medicine, Doha, Qatar; 3 Clinical Pharmacy Division, Hamad Medical Corporation, Doha, Qatar; 4 Brown University, College of Medicine, New York, USA; 5 Department of Internal Medicine, Hamad Medical Corporation, Doha, Qatar; 6 Department of Internal Medicine, Qatar University College of Medicine, Doha, Qatar

**Keywords:** Diagnostic markers, Skin diseases, Biomarkers

## Abstract

Drug reaction with eosinophilia and systemic symptoms (DRESS) syndrome is a rising morbidity amongst hospitalized patients. Whilst clinical protocols for the management of individual DRESS cases have been well established, determination of potential prevention of these cases by utilizing novel “avoidability” tools has remained unexplored. This retrospective study reviewed records of patients who presented to the emergency department of Weill Cornell Medicine-affiliated Hamad General Hospital, Doha Qatar with suspected DRESS syndrome. These cases were independently adjudicated (utilizing the RegiSCAR, and JSCAR tools) as DRESS-drug pairs by a team of two clinical pharmacists and two General Physicians. They were then rated for potential avoidability with the Liverpool adverse drug reactions avoidability tool (LAAT) by the same team of raters. A total of 16 patients satisfied RegiSCAR criteria for DRESS syndrome. The mean age of the study population was 41.5 years (SD ± 13.3). The study population was predominantly male (n = 12; [75%]). The median latent period from drug ingestion to clinical presentation was 14 days (interquartile range [IQR] 6.5, 29). The median RegiSCAR and J-SCAR scores were 6 (IQR 5, 6.8), 5 (IQR 4, 5.8) respectively. Utilizing the LAAT, about 60% of the DRESS syndrome-drug pairs were rated as “avoidable” (“probable” or “definite”). The overall Krippendorf’s alpha with the LAAT was 0.81 (SE 0.10, CI 0.59–1.00); with an intraclass correlation coefficient (ICC) of 0.90 (CI 0.77, 0.96.). In a randomly selected cohort of DRESS syndrome-drug pairs, a significant proportion was potentially avoidable (“possibly” and “definitely”) utilizing the LAAT. This will need validation by larger sample-sized prospective studies utilizing the updated LAAT proposed by this study.

## Introduction

First recognized in 1937, drug rash with eosinophilia and systemic symptoms (DRESS) syndrome is an idiosyncratic reaction to a range of drugs; often leading to systemic manifestations including rash, fever, lymphadenopathy, eosinophilia, renal/hepatic injury^1^. The temporal profile of its presentation is variable, but is suggested to range between 1 and 8 weeks^1^. The reported prevalence of clinically significant DRESS syndrome has been estimated to range between 1 in 1000, and 1 in 10,000 exposures^1–4^, with no discernible gender bias^5^. Amongst the range of drugs thus far identified to cause DRESS syndrome, includes allopurinol (32%), phenytoin (18%), dapsone (17%), vancomycin (39%), penicillin (13%) amongst others^3,6^. Multiple clinical adjudication tools and algorithms have been developed and subsequently validated to aid clinicians in the determination of the likelihood of DRESS in patients who present with clinical features that raise suspicion. These tools include the European Registry of Severe Cutaneous Adverse Reactions (RegiSCAR)^1^, and the Japanese Research Committee on Severe Cutaneous Adverse Reaction (J-SCAR)^2^; both of which have been exhaustively validated and are currently clinical use. DRESS syndrome confers significant morbidity and sometimes unacceptable excess mortality, with rates of between 5 and 10% reported in some series^1,7^. Despite these clinical burden, current attempts have focused primarily on management of each index case with paucity of work on its potential preventability. The most recent Spanish Guidelines for diagnosis, management, treatment, and prevention of DRESS Syndrome made recommendations centered around added predictive values of specific pharmacogenetic makers (such as single nucleotide polymorphisms) encoding implicated drugs^8^. Recently, there has been a rising interest in the determination of the avoidability of adverse drug reactions (ADR) in the general population using novel “avoidability” determination algorithms/tools^9–12^. One of such tools is the Liverpool adverse drug reaction avoidability tool (LAAT)^9^. Despite recent utility of this tool in the determination of ADR in other drug-related clinical risks^9,11,12^, its potential impact on adjudication of DRESS syndrome-drug pairs have remained unexamined. The potential utility of avoidability determination in the prevention of morbidities related to ADR (such as DRESS syndrome) are enormous; including positive impact on and supporting clinical decision framework around therapeutic commissioning of specific drugs associated with increasing DRESS association and demonstrable preventability. Therapeutic commissioning which involves the assessment of needs, identification, appraisal (both clinical and economic), and recommendation of specific therapeutic agents for inclusion into local, regional, and international treatment formularies (often by drug and therapeutic committees) presents excellent targets for interventions that aim to address both the causes and potential avoidability of ADRs. In this study, we have for the first time explored the potential utilization of the LAAT tool in the setting of DRESS syndrome to ascertain its utility in determining the avoidability of this syndrome. The outcome of our exploratory work could further assist in the development of clinical guidelines and policies that are more proactive and focused on prevention rather than the current reactive paradigm focused primarily on management of individual patients.

## Methods

This retrospective study was comprised of patients presenting with suspected DRESS syndrome to the emergency department (ED) of Weill Cornell Medicine-affiliated Hamad General Hospital (HMC), Doha Qatar as part of an observational adverse drug reactions cohort. From this population, 16 suspected cases presenting between March 2018 and April 2020 were initially adjudicated (by their primary physicians and dermatologists) for potential diagnosis of DRESS syndrome and then selected as part of this current study’s cohort. Socio-demographic, clinical, and laboratory parameters of the study participants were abstracted from an online patient record system (Cerner) to a Microsoft excel data collection database. Variables of interest includes age, gender, ethnicity, comorbidities, implicated drugs, drug latent periods, as well laboratory parameters required for completing the RegiSCAR and JSCAR algorithms. Implicated drugs were identified by raters based on the likelihood of causing DRESS syndrome as reported in the literature considering the time factor in the patient’s hospital course. Two independent assessors determined the most likely offending agent by reviewing the patient's chart and any discrepancies were resolved by discussion, revision of the time frame, and dermatology opinion.

The study protocol and all relevant study documentation were reviewed and approved by Hamad Medical Corp (HMC) institutional review board. All patients provided informed consent to participate in the study. All methods were performed in accordance with all relevant guidelines and regulations.

### Avoidability determination

**Figure 1:**
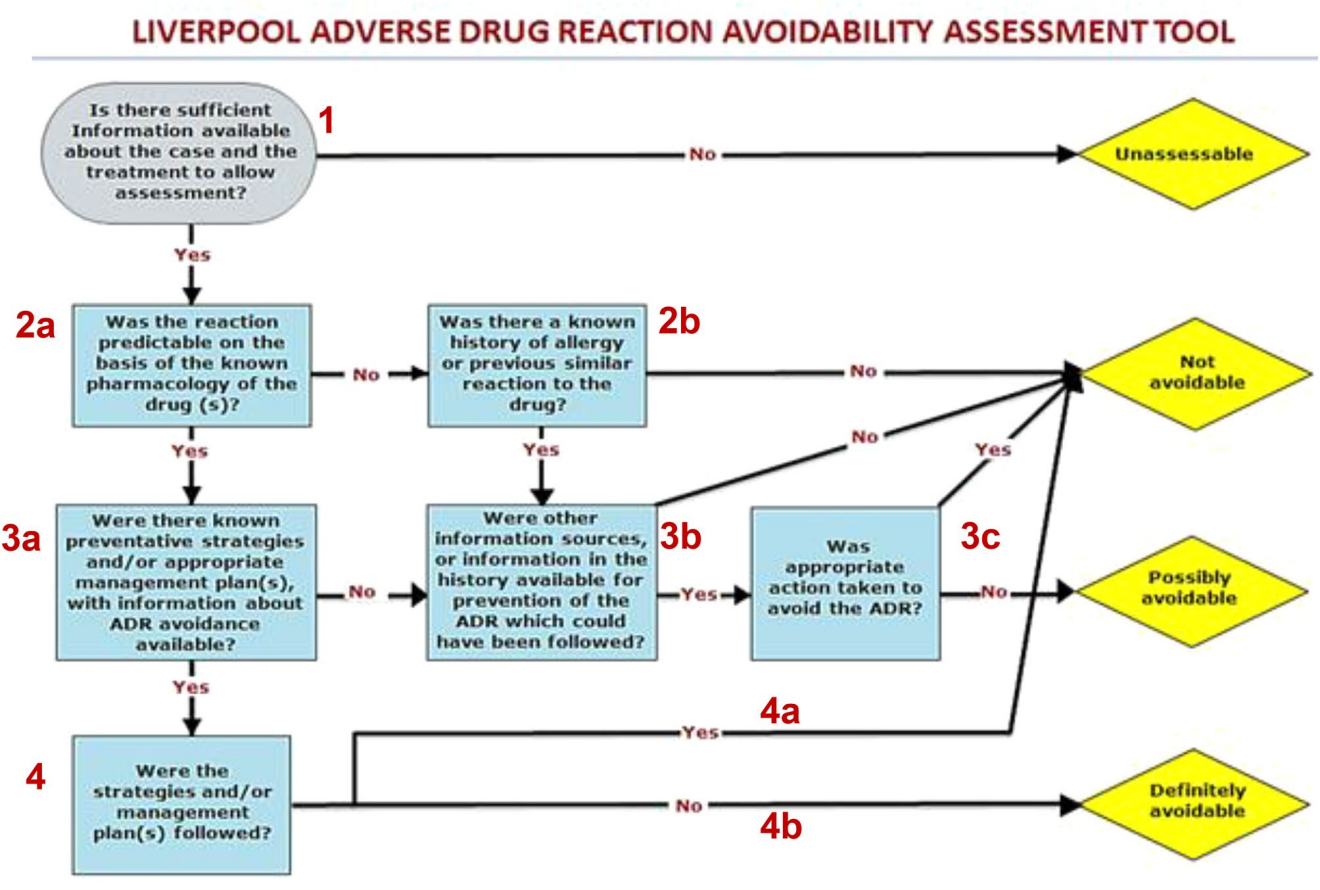
The Liverpool ADR avoidability assessment tool (LAAT). Adapted from Ref.^9^.

To determine potential avoidability of ADRs, two independent rating pairs (2 Clinical Pharmacists and 2 General Physicians) utilized the LAAT tool to score DRESS syndrome-drug pairs (Fig. 1). Avoidability of DRESS syndrome outcomes were reported as “definitely avoidable”, “possibly avoidable”, “not avoidable” and “unassessable”.

.

### Statistical analysis

Ordinal outcomes from diagnostic, and avoidability tools are represented as numbers (%), with their pairwise interrater agreement proportions, Krippendorf’s kappa statistics with 95% confidence intervals (CI), and intraclass correlation coefficients (ICC). To determine agreement across multiple assessors, we calculated and compared the pairwise scores with a global kappa score. All statistical analyses were conducted with Stata, (StataCorp. 2019. Stata Statistical Software: Release 16. College Station, TX: StataCorp LLC).

### Case definition and ascertainment

#### DRESS syndrome

- Patients are adjudged as having DRESS syndrome if RegiSCAR score ≥ 5. Two independent rating pairs (2 Clinical Pharmacists and 2 General Physicians) initially assessed the likelihood of DRESS syndrome diagnosis utilizing DRESS syndrome scoring tools (RegiSCAR and J-SCAR)^1^ prior to avoidability determination. The ordinal outcomes for the RegiSCAR tool were reported as “no case”, “possible case”, “probable case” and “definite case”^1^; while that for the JSCAR tool were reported as “consider other diagnosis”, “atypical DRESS”, and “typical DRESS”^2^
- Extreme agreement (EA) between rating pairs was defined as a situation where both raters scored a DRESS syndrome-drug pair to the same ordinal outcome.
- Extreme disagreement (ED) between rating pairs was defined as a situation where a rater adjudicates a DRESS syndrome-drug pair outcome as unassessable” whilst the other rater assessed it as any of the three other outcomes (“not avoidable”, “possibly avoidable”, or “definitely avoidable”)^10^.
- Kappa values of ≤ 0.20, 0.21–0.40, 0.41–0.60, 0.61–0.80, and 0.81–1 represented a slight, fair, moderate, substantial, and almost perfect agreement, respectively^13^.

## Results

The baseline characteristics of the study population (*n* = 16) are shown in Table 1. A total of 16 patients satisfied RegiSCAR criteria for DRESS syndrome. The mean age of the study population was 41.5 (SD ± 13.3) years and was comprised of disproportionately male subjects (n = 12; [75%]). The median RegiSCAR and J-SCAR scores were 6 (IQR 5, 6.8), 5 (IQR 4, 5.8) respectively. The comparative interrater agreement (IRR) proportion between the four different raters (two General physicians and two clinical pharmacists) utilizing the RegiSCAR and J-SCAR tools were 0.4984 (CI 0.18–0.82), 0.31 (CI − 0.07 to 0.71) respectively. The median latent period from drug ingestion to clinical presentation was 14 days (IQR 6.5, 29). Other estimates of key laboratory determinants including serum eosinophils are given in Table 1.

**Table 1:** Baseline characteristics of the study population (N = 16).

Variable description	*N* = 16	Reference values
Age (years) Mean (SD)	41.5 (13.3)	–
Gender (male) N (%)	12 (75)	–
AST median (IQR) U/L	50 (27, 124)	5–40
ALT (U/L) Median (IQR)	56 (22, 160)	7–56
ALP (U/L) Median (IQR)	115 (97, 145)	44–147
Albumin (g/L) Median (IQR)	34 (25, 38)	35–50
Neutrophils (× 10^3^ cells/µL) Median (IQR)	2.5 (23.5, 67)	2–7
Eosinophils (× 10^3^ cells/μL) Median (IQR)	4.5 (2.4, 7.5)	0.0–0.5
EGFR*^7^ (mL/min/1.73 m^2^ Median (IQR)	60 (60, 107)	> 60
Drug latent period (days) Median (IQR)	14 (6.4, 29.2)	–
Extent of skin rash (> 50%)	9/16	
**Internal organ involvement**		
Liver (%)	25	
Kidneys (%)	37.5	
RegiSCAR Median (IQR)	6 (5, 6.8)	
J-SCAR Median (IQR)	5 (4, 5.8)	
Proportion of EBV positivity (%)	31.2	

*SD* Standard deviation, *AST* aspartate aminotransferase, *ALT* alanine aminotransferase, *ALP* alkaline phosphatase, *PT* prothrombin time, *IQR* interquartile range, *EGFR* estimated glomerular filtration rate.

### DRESS syndrome avoidability outcomes

The LAAT tool utilization resulted in a total of 60 outcome decisions. Of these, 6.7% were rated as “unassessable”, 33.3% were rated as “not avoidable”. Significantly, 60% of the decisions were rated as “avoidable” (“probable” or “definite”). The overall Krippendorf’s alpha with the LAAT was 0.81 (SE 0.10, CI 0.59–1.00). The intraclass correlation coefficient (ICC) utilizing the LAAT tool was 0.90 (CI 0.77, 0.96). The proportion of exact agreement between raters was 86.7%. (95% CI 0.71–1.00). The drugs most associated with DRESS syndrome were beta lactam antibiotics 43.7% (*n* = 7), Allopurinol 25% (*n* = 4), and NSAIDS 18.7% (*n* = 3). Table 2 gives a contextual explanation for why some DRESS-drug pairs were potentially preventable.

**Table 2:** Depicting the probable reasons for potential preventability of various drugs (from examination of individual LAAT scores).

Implicated drugs	“Avoidable”	“Not avoidable”	Comments Potential reasons for avoidability
Amoxicillin	3	0	Exceeded duration of treatment
Allopurinol	–	4	
Ibuprofen	2	–	Uncertain indication/over the counter prescription
Naproxen	–	1	–
Ceftriaxone	–	3	–
Piperacillin/tazobactam	1	–	Uncertain indication/inadequate determination of patient therapeutics risks prior to commencement (including drug allergies in the past)
Desloratadine	–	1	–
Dasatanib	1		Inadequate determination of patient therapeutics risks prior to commencement (including drug allergies in the past)

## Discussion

To our knowledge, this study represents the first examination of the potential preventability of DRESS syndrome-drug pairs by utilizing the LAAT. We found about 60% of DRESS syndrome-drug pairs were potentially avoidable (“probable” or “definite”). The exact agreement proportions between the raters in terms of avoidability was about 80%; which will indicate excellent agreement. Our report is consistent with the agreement proportions reported by the original developers of the tool^9^, as well subsequent validation studies^10,11,14^. The proportionately higher interrater agreement and avoidability proportions (0.80) reported from our study compared to that by both Louise et al.^9^ and Danjuma et al.^10^ may be due to the phenotype of the clinical risks the tool (LAAT) was attempting to adjudicate. The fact that DRESS syndrome has a distinct cutaneous clinical phenotype may have improved the degree of certainty in both the interrater agreement as well as avoidability determination outcomes compared to that seen with other ADR’s (such as drug induced liver injury^11^. We do concede that some forms of DRESS syndrome are idiosyncratic in manifestation and may confound attempts at adjudicating their potential avoidability determination. However what proportion of this is attributable the latter category in our current sample population is difficult to ascertain (largely because of our small sample size).

Our initial use of both RegiSCAR and J-SCAR tools was to allow a more robust determination of the diagnosis of the likelihood of DRESS syndrome in the study subjects. This is to ensure a more rigorous adjudication of potential avoidability afterwards. Although an exhaustive comparative analyses of the determinative values of the RegiSCAR and JSCAR tools is not within the remit of this study, but we found it interesting that the interrater agreement proportions were lower with the JSCAR (0.31) compared to the Regi-SCAR tools (0.50). This represents “slight” agreement between the raters with the JSCAR versus “good” agreement in the RegiSCAR tool^13^. The limitations of the J-SCAR as a DRESS syndrome adjudication tool have exhaustively been discussed elsewhere including the need for demonstration of Human Herpes 6 reactivation (HHV-6)^15^. The key novelty and strength of our study lies is its seminal attempt at examination of potential avoidability of DRESS syndrome by utilizing the LAAT. Additionally, the initial determination of likelihood of diagnosis of DRESS with two of the most widely validated tools confers added rigor to the adjudication process. We deliberately made a comparison between clinical pharmacist and the General physicians, as they represent distinct specialties that are comparatively more likely to encounter this morbidity in their daily practice. Despite the relatively high agreements proportions between the rating pairs, we have identified several factors which may have contributed to disagreements in the adjudication of potential avoidability of DRESS syndrome-drug pairs. Some of these includes; differences (amongst raters) in the background pharmacological understanding of the implicated drugs vis-à-vis the various reported phenotypes of their adverse events profile; varying interpretation of the path of the LAAT that was open to subjective interpretation (such as questions 2a and 4 in Fig. 1) amongst others.

**Figure 2:**
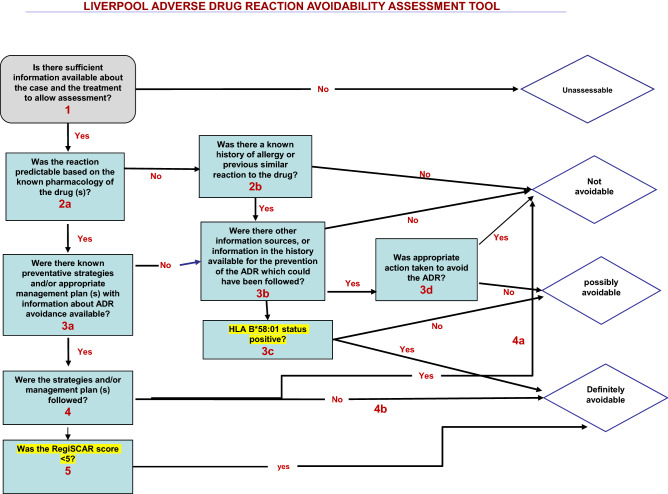
Schematic representation of the Liverpool adverse drug reactions avoidability tool showing the path of determination of avoidability of DRESS syndrome-Drug pairs (adapted from Bracken et al. ^9^).

Our study was limited by its small sample size (given its rarity) as well as the same constraints associated with the use of such data schemes; these includes the potential of the LAAT to misclassify ADRs that are predominantly hypersensitivity related (Type B reactions); generic design of the LAAT tool, which on one hand allows clinicians to determine the avoidability of a wide range of adverse events, but opens up others such as skin dermatosis to wide range of interpretations which potentially could confound our results. Indeed, it is consequent upon this that, we propose modification of the LAAT tool to incorporate the RegiSCAR score into its avoidability determination path for subsequent studies in this area (Fig. 2). Such incorporation of RegiSCAR score into the LAAT will probably hold true for other delayed type reactions (such as Steven Johnson Syndrome [SJS], toxic epidermal necrolysis [TENS], and overlap syndromes [SJS/TENS]) as well, but this will need exploration by future prospective studies. Additionally, since a significant proportion of DRESS cases have thus far been attributable to Allopurinol^3^, adding genetic markers defining its involvement such as HLA B*58:01^16^ to LAAT may enhance its determinative utility, but this will need ascertainment by future studies.

.

The proportion of potentially avoidable DRESS syndrome outcomes from our report if validated by larger sample sized systematic diagnostic studies will have implication for review of therapeutic commissioning and or practices around the implicated drugs. For example, drugs with consistently high adverse reaction avoidability estimates may be subjected to more rigorous evaluation (compared to their alternatives) before commissioning into formularies (local or national). Additionally, avoidability estimates will assist in devising strategies on how to reduce potential ADRs (especially in situations where there are no veritable substitutes for the therapeutic agents in question). We envisage the LAAT as an additional risk stratification tool amongst other algorithms and scores aimed at reducing the ever-growing pharmacovigilance burden in the population.

## Conclusion

In a randomly selected sample of DRESS syndrome-drug pairs, the LAAT tool showed a significant proportion that were potentially avoidable (“possibly” and “definitely”). This will need validation by large sample sized studies utilizing the updated LAAT tool proposed by this study.

## Data Availability

All data relating to this work is available from the corresponding author on reasonable request.
